# Nanostructured Fe_2_O_3_/Cu_*x*_O heterojunction for enhanced solar redox flow battery performance†

**DOI:** 10.1039/d4ta06302c

**Published:** 2024-11-27

**Authors:** Jiaming Ma, Milad Sabzehparvar, Ziyan Pan, Giulia Tagliabue

**Affiliations:** a Laboratory of Nanoscience for Energy Technologies (LNET), STI, École Polytechnique Fédérale de Lausanne 1015 Lausanne Switzerland giulia.tagliabue@epfl.ch

## Abstract

Solar redox flow batteries (SRFB) have received much attention as an alternative integrated technology for simultaneous conversion and storage of solar energy. Yet, the photocatalytic efficiency of semiconductor-based single photoelectrodes, such as hematite, remains low due to the trade-off between fast electron hole recombination and insufficient light utilization, as well as inferior reaction kinetics at the solid/liquid interface. Herein, we present an α-Fe_2_O_3_/Cu_*x*_O p–n junction, coupled with a readily scalable nanostructure, that increases the electrochemically active sites and improves charge separation. Thanks to light-assisted scanning electrochemical microscopy (photo-SECM), we elucidate the morphology-dependent carrier transfer process involved in the photo-oxidation reaction at an α-Fe_2_O_3_ photoanode. The optimized nanostructure is then exploited in the α-Fe_2_O_3_/Cu_*x*_O p–n junction, achieving an outstanding unbiased photocurrent density of 0.46 mA cm^−2^, solar-to-chemical (STC) efficiency over 0.35% and a stable photocharge–discharge cycling. The average solar-to-output energy efficiency (SOEE) for this unassisted α-Fe_2_O_3_-based SRFB system reaches 0.18%, comparable to previously reported DSSC-assisted hematite SRFBs. The use of earth-abundant materials and the compatibility with scalable nanostructuring and heterojunction preparation techniques offer promising opportunities for cost-effective device deployment in real-world applications.

## Abstract 2

An α-Fe₂O₃/CuxO p–n junction SRFB achieves 0.46 mA cm⁻² photocurrent density, >0.35% STC efficiency, and 0.18% SOEE, leveraging earth-abundant, scalable materials for enhanced charge separation, electrochemical activity, and practical deployment.

## Graphical abstract

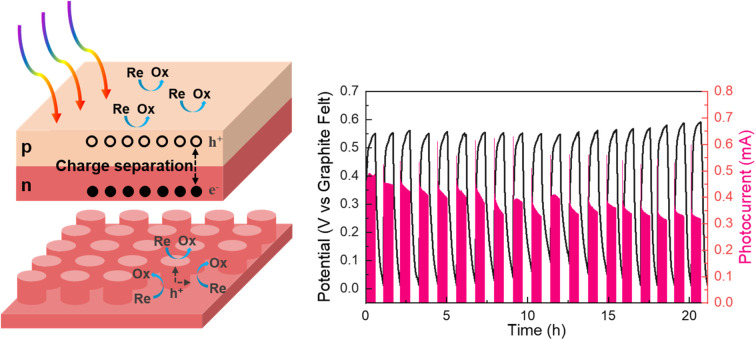

## Introduction

Solar energy conversion offers a promising solution to meet the steadily increasing energy demand sustainably. Through the combination of photoelectrochemical cells (PEC) and redox flow batteries (RFB), solar energy can be efficiently converted and stored as chemical fuels by oxidizing or reducing various redox couples.^1–3^ The success of this all-in-one solar redox flow battery (SRFB) mainly depends on the design of the cell structure^4,5^ and the development of high-performance photoelectrodes.^6,7^

Theoretically, the maximum solar-to-chemical (STC) efficiency of a single photoelectrode SRFB system can reach 16–18% if the bandgap of the absorber material is within 1.4–2 eV and the thermodynamic cell voltage is around 0.9 V and 0.7 V.^8^ Yet, practical realizations have not surpassed 3.9% STC efficiencies^9^ with limited upscale fabrication. This is related to band-alignment constraints for SRFB photocharging up to high states of charge, which favor wide-bandgap materials with limited solar light absorption.^10^ Additionally, low efficiency of charge separation dramatically reduces the performance of real devices. STC conversion efficiencies up to 21.1% have been instead achieved by exploiting complex device structures (*e.g.* dual photoelectrodes^11^) or more expensive tandem photoelectrodes (*e.g.* multi-junction solar cells^12,13^). Thus, the development of photoelectrodes with low cost and scalable manufacturing necessitates further investigation.^14–18^

Hematite (α-Fe_2_O_3_) is a promising photoanode material due to its stability, non-toxicity, low cost, abundance on Earth, and attractive band gap^19,20^ (1.9–2.2 eV). However, its performance is hindered by the trade-off between light-absorption and charge separation/transport, due to the short hole diffusion length^21^ and poor charge carrier conductivity.^6,22^ Hematite thin films^23,24^ have been used to reduce the charge carrier diffusion distance to the electrode/electrolyte interface at the expense of complete light absorption (a thickness of 40–100 nm is needed to absorb 450–550 nm light^25^). An effective approach to overcome this trade-off is the use of nanoengineered structures, which can shorten the charge carrier transfer length,^26–28^ increase the electrochemically active surface area,^29^ achieve light trapping,^30^ and induce optical resonances within the active photocatalyst material itself.^31^ Additionally, properly engineered heterojunction photoelectrodes, *e.g.* based on a p–n junction,^32^ can further improve the spatial separation of photogenerated electron–hole pairs,^33^ enhancing the photocatalytic activity. In the context of water-splitting or photosynthetic devices, a significant amount of effort has been devoted to proposing and designing heterojunctions aimed at efficiently extracting photo-holes from α-Fe_2_O_3_ catalysts.^19,34–37^ Yet, this approach has not been thoroughly explored in semiconductor-based SRFBs, despite major advantages in enabling higher state-of-charge and higher voltages during photocharging and discharging, respectively. Overall, nanoengineering and heterojunction design have a large untapped potential for improving single photoelectrode SRFB PEC performance.

**Fig. 1:**
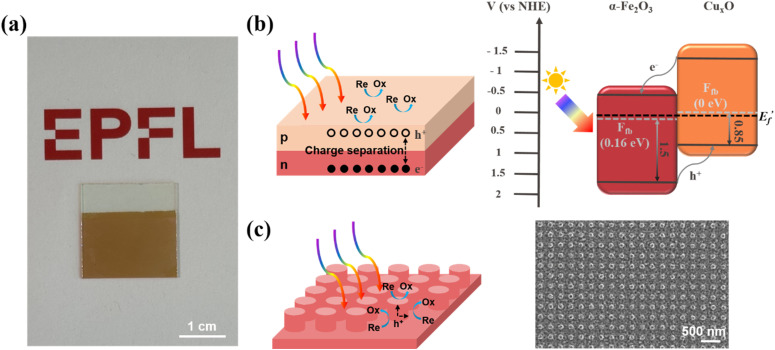
(a) Digital figure of a P/Cu_*x*_O sample (5 cm^2^), obtained by synergistically combining heterojunction engineering with large-area nanofabrication (nanosphere lithography); (b) schematic of a planar p–n junction and its contribution to electron–hole separation (left) as well as quantitative band alignment of the as prepared α-Fe_2_O_3_ and Cu_*x*_O (right, 
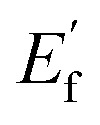
 is the Fermi level after contact); (c) schematic a nanostructured photoanode and its improved hole collection (left) and SEM of one of the studied α-Fe_2_O_3_ nanopillars (right, period of 300 nm and diameter of 150 nm).

In this work, we present a scalable, nanostructured α-Fe_2_O_3_/Cu_*x*_O p–n junction and demonstrate its largely improved unassisted photocharging of an integrated solar redox flow battery (Fig. 1a). First, α-Fe_2_O_3_/Cu_*x*_O films with varying thicknesses were systematically investigated to elucidate the impact of the p–n junction on the photoelectrochemical performance (Fig. 1b). Concurrently, light-assisted scanning electrochemical microscopy (photo-SECM) was employed to reveal enhanced charge separation in α-Fe_2_O_3_ nanopillar arrays (Fig. 1c). Finally, guided by the results from SECM, the optimized α-Fe_2_O_3_ nanostructure was integrated with the p–n junction strategy to enhance charge carrier separation while improving electrochemically active sites, thus resulting in a high performance photoanode. Our SRFB, featuring the nanostructured α-Fe_2_O_3_/Cu_*x*_O p–n junction, demonstrates record values of unassisted photocurrent (0.46 mA cm^−2^), along with STC efficiency ∼ 0.35% and SOEE ∼ 0.18%, comparable to solar cell-assisted hematite-based devices (Table S1†). Overall, this α-Fe_2_O_3_-based SRFB shows a stable photocharge–discharge cycling performance and presents opportunities to drive real-world deployment of more cost-effective devices.

## Experimental section

### Sample preparation

#### Synthesis of α-Fe_2_O_3_/Cu_*x*_O film photoanodes (Fig. S1a, ESI†)

RF magnetron sputtering was used to sputter iron thin films (15, 30 and 50 nm) on Indium Tin Oxide (ITO) glass under the protection of argon gas. Subsequently, copper thin films with different thickness (15, 30 and 45 nm) were sputtered on the iron films under the same condition. The Fe/Cu films were then immersed in 4 M NaOH (1 h at 80 °C, then 20 h at room temperature) to get Fe/Cu_2_O film in accordance with previous reports.^38^ Next, all the samples were annealed at 645 °C under air for 10 minutes with the ramping of 5 °C min^−1^ to obtain α-Fe_2_O_3_/Cu_*x*_O (named F-Cu_*x*_O) photoanodes. The control samples, named 15F, 30F, and 50F, consisted of iron films with thicknesses of 15 nm, 30 nm, and 50 nm, respectively, without copper coating or NaOH treatment, all subjected to the same annealing process, which turns them into hematite films.

#### Synthesis of α-Fe_2_O_3_/CuO film photoanodes

Iron thin film (15 nm) was sputtered on Indium Tin Oxide (ITO) glass by using the same method mentioned above. Subsequently, copper thin (30 nm) was sputtered on the iron film under the same condition. Then, the sample was annealed at 645 °C under air for 10 minutes with the ramping of 5 °C min^−1^ to obtain α-Fe_2_O_3_/CuO (named 15/30F-CuO) photoanodes.

#### Synthesis of α-Fe_2_O_3_ nanopillar array (Fig. S1b, ESI†)

Iron thin film (30 nm) was sputtered on Indium Tin Oxide (ITO) glass by using the same method mentioned above. The Fe nanopillars were then made by e-beam lithography and ion beam etching. ZEP-520A (50%) was spin coated at 4000 rpm rate (∼120 nm) on a precleaned iron sample, followed by baking at 180 °C for 5 minutes. E-beam was used to pattern nanostructures on photoresist and the sample was subsequently developed in amyl-acetate solution for 1 minute. The desired Fe nanopillars (300 nm periodicity; 100, 150 and 200 nm in diameter) was then fabricated by ion beam etching with 1.1 nm s^−1^ etching speed. The as-prepared Fe nanostructure consists of nanopillars that are 25 nm in height, with a continuous Fe layer that is 5 nm thick at the bottom. The sample was cleaned *via* oxygen plasma (150 sccm O_2_, 200 W) for 10 s, followed by annealing at 645 °C under air for 10 minutes to obtain α-Fe_2_O_3_ nanopillar array (named P100, P150 and P200).

#### Synthesis of nanostructured α-Fe_2_O_3_/Cu_*x*_O photoanode (Fig. S1c, ESI†)

Iron thin films (30 nm) were sputtered on Indium Tin Oxide (ITO) glass as mentioned above. Polystyrene (PS) nanospheres (Microparticles GmbH) with average diameter of 300 nm were then coated on top of iron film *via* Langmuir–Blodgett (LB) technique as a monolayer.^39^ Oxygen plasma (800 sccm O_2_, 300 W) was used to reduce the PS nanospheres to a diameter ranging from 150 to 180 nm. The PS etching speed is around 7.5 nm min^−1^. Subsequently, similar Fe nanopillars with Fe thin film (∼5 nm) was obtained *via* ion beam etching and beads removing process.^40^ 30 nm copper film was then sputtered on the Fe nanostructure. The same NaOH treatment and annealing process as mentioned above were performed to get the nanostructured α-Fe_2_O_3_/Cu_*x*_O (named P/Cu_*x*_O) photoanode. The bare nanostructured α-Fe_2_O_3_ (named P) without copper coating and NaOH treatment was also fabricated as a control sample *via* the same method.

The summary of the sample preparation process is shown in Table S2.†

### Material characterization

The morphology and crystal structure of photoanodes were characterized by a scanning electron microscope (Zeiss Gemini SEM 300) and X-ray diffractometer (XRD, Rigaku Synergy-I single crystal). Ultraviolet photoelectron spectroscopy (UPS) was performed using a PHI VersaProbe II scanning XPS microprobe (Physical Instruments AG, Germany) equipped used with He(i) and He(ii) UV source.

### Optical measurements

The UV-vis test for α-Fe_2_O_3_/Cu_*x*_O film and nanostructured α-Fe_2_O_3_/Cu_*x*_O were performed under a solar simulator (Newport 66984-300XF-R1 Xe lamp) with an AM 1.5 G filter as the light source, using a monochromator (Newport, CS260B-2-MC-A) connected with an integrating sphere (Newport, 819D-IS-5.3). The absorption data was obtained following our previous work.^41^

For the α-Fe_2_O_3_ nanopillars, an inverted microscope (Nikon Eclipse Ti2) was used in combination with a grating spectrometer (Princeton Instruments Spectra Pro HRS-500) equipped with a Peltier-cooled 2D CCD detector (Princeton Instruments PIXIS 256) to record reflection (*R*) and transmission (*T*) spectra. The absorption (*A*) was determined as *A* = 1 − *R* − *T*. The detailed process is in accordance with previous reports.^42,43^

### Photoelectrochemical measurements and photocharge–discharge

Linear Sweep Voltammetry (LSV), photocurrent density–time (*j*–*t*) and Electrochemical Impedance Spectroscopy (EIS) were used to evaluate the photoelectrochemical performance of different photoanodes and were recorded on a Biologic SP-300 potentiostat. LSV were tested both in dark and under illumination with a scan rate of 10 mV s^−1^; *J*–*t* was recorded without bias and the photo response signal were obtained with 20–20 s light on/off. EIS analyses were carried out at a perturbation amplitude of 10 mV with the frequency ranging from 0.01 Hz to 10 kHz. EC-Lab software was used to fit the measured EIS results. All tests were carried out by using a two-electrode configuration of the SRFB device^44^ (one photoanode in anolyte and one 39 AA carbon felt in catholyte, as shown in Fig. S2†) under 1 sun illumination (AM 1.5 G filter, 100 mW cm^−2^). All samples were back-illuminated through the ITO glass, and the illuminated area is 0.785 cm^2^.

The photocharge–discharge behavior of our solar redox flow batteries was demonstrated *via* three electrodes integrated SRFB and was recorded by two potentiostats. During photocharging process, potentiostat 1 (SP-300) was connected to the PEC part to monitor the photocurrent, while potentiostat 2 (CHI 760E), connecting the RFB part, measured the evolution of the cell potential. During the discharging process, the solar simulator and potentiostat 1 were turned off, while a discharging current of 0.4 mA was applied by potentiostat 2 to RFB part until the cell potential reached 0 V.

During all these tests, the electrolyte recirculation was guaranteed by using two peristaltic pumps (30 ml min^−1^), both the anolyte (0.2 M Na_4_Fe(CN)_6_/1 M NaOH) and catholyte (0.1 M 2,7-AQDS/1 M NaOH) volume of the running system were 4 ml.

### Scanning electrochemical microscopy

Light-assisted scanning electrochemical microscopy (photo-SECM) was implemented to investigate the photocatalytic activity of micro-array structures (100 × 100 μm^2^) of α-Fe_2_O_3_ nanopillars, using a previously described home-built instrument.^42^ Briefly, a home-built SECM was coupled with an inverted optical microscope (Nikon Eclipse Ti2) and a white light source (Energetiq EQ-99X-FC LDLS) for back-illumination of the structures. A three-electrode configuration was employed with Pt and Ag/AgCl as counter and reference electrodes, and Pt ultra-microelectrode (Pt UME) tip as the working electrode. The structured samples were unbiased and grounded. Tip to substrate distance was controlled by monitoring the tip current during its fine approach towards the substrate and lifting up the tip by 3 μm higher than the distance corresponding to 30% change from the bulk state. All experiments were performed in a 4 mM K_4_Fe(CN)_6_^4−^ and 0.4 M KOH solution, using a 1.2 μm radius UME tip (RG value = 14.5) biased at a reductive 0 V *vs.* Ag/AgCl tip potential, and modulating a ∼90 μm diameter collimated light beam having 80 mW m^−2^ power density.

### Numerical simulation

Electromagnetic simulations were performed using the RF module of COMSOL Multiphysics v6.1 to obtain the absorption spectra of α-Fe_2_O_3_ nanopillars and α-Fe_2_O_3_ film. For α-Fe_2_O_3_ nanopillars, a 3D unit cell model with periodicity of 300 nm, consisting of one α-Fe_2_O_3_ nanopillar (50 nm in height) on top of 10 nm α-Fe_2_O_3_ film/100 nm ITO film/fused silica substrate surrounded with a top layer of air, was simulated by setting the diameter of nanopillars from 100 nm to 250 nm with 10 nm step size. For α-Fe_2_O_3_ film simulations, a similar 3D unit cell model without the α-Fe_2_O_3_ nanopillar was performed by varying the film thickness from 20 to 180 nm with 10 nm step size. In both cases, perfect magnetic conductor and perfect electric conductor boundary conditions were used at the side walls of the unit cell. A port boundary condition was used at the bottom of the unit cell. The back illumination was applied with a normal incident plane wave (300–850 nm) with electric field polarization perpendicular to the film plane as well as recording the reflected wave. At the top of the unit cell, a second port boundary condition without excitation was used to record the transmitted wave. The refractive indices for α-Fe_2_O_3_ and ITO were taken from literatures^45,46^ Then the absorbed power was calculated by volume integration of the electromagnetic power loss density over the α-Fe_2_O_3_ volume.

## Results and discussion

α-Fe_2_O_3_/Cu_*x*_O heterojunctions were fabricated *via* a facile and scalable method to enhance electron/hole separation in SRFB photoanodes. Specifically, after sputtering 15 nm Fe and between 15 nm and 45 nm Cu onto ITO, a NaOH treatment was used to convert Cu to Cu_2_O.^38^ Subsequently, annealing of the as-prepared composite leads to the formation of α-Fe_2_O_3_–Cu_2_O–CuO, concisely referred to α-Fe_2_O_3_/Cu_*x*_O (see Experimental section). In the following, samples are named according to the thickness of the initial Fe and Cu film (*e.g.* 15/30F-Cu_*x*_O for the heterostructure). We note that an approximately 2 fold expansion is expected for conversion from Fe to Fe_2_O_3_ (ref. 47) while a significant reduction in thickness, from 30 nm to 15 nm, is observed for the Cu to Cu_*x*_O conversion duo to the partially dissolution of Cu into NaOH^38^ (Fig. S3, ESI†). As a control sample, we use a 15 nm Fe film that is annealed under the same conditions without NaOH treatment (sample 15F, see Experimental section).

Fig. 1b shows the estimated band alignment for our α-Fe_2_O_3_/Cu_*x*_O heterojunction, obtained by combining the measured bandgap from Tauc plot, work function from the Mott–Schottky technique and the valence band maxima from UPS (Fig. S4, ESI†). This is indeed crucial to assess the energy levels compatibility between the electrode/electrolyte. We confirmed that Cu_*x*_O is a p-type semiconductor and that a p–n junction is formed at the α-Fe_2_O_3_/Cu_*x*_O interface. While this is expected to promote charge separation, and hence the photoelectrochemical performance of the photoanode,^33^ it can reduce the possible theoretical discharge cell voltage (Fig. 1b), as the oxidation and reduction potentials of the chosen redox couples must lie within the p-type semiconductor valence band and the n-type semiconductor conduction band energies.^33^ We chose Fe(CN)_6_^4−^/Fe(CN)_6_^3−^ as the anolyte (0.60 V *vs.* normal hydrogen electrode (NHE)) and AQDS/AQDS^2−^ (−0.14 V *vs.* NHE) as the catholyte to evaluate the performance of the designed α-Fe_2_O_3_-based photoanodes.^5^

**Fig. 2:**
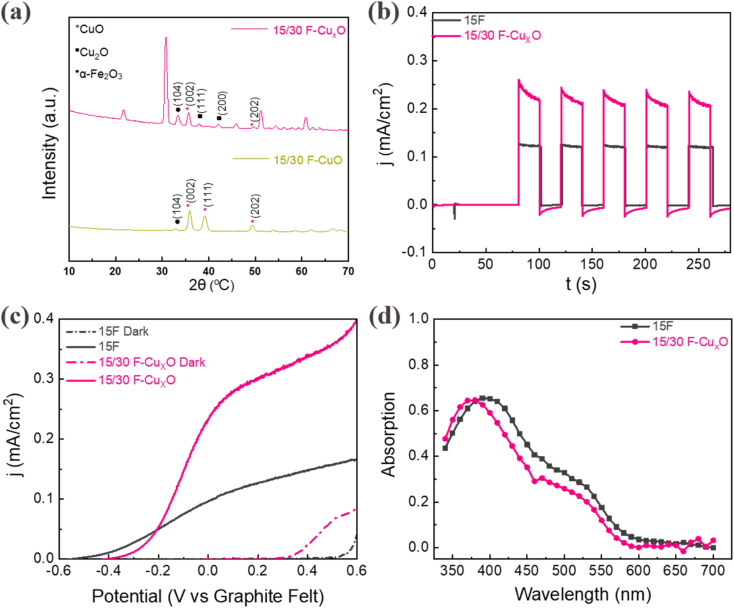
(a) XRD of different photoelectrodes; (b) photoresponse behavior for 15/30F-Cu_*x*_O and 15F with 20 s light on–off; (c) linear sweep voltammetry curve for 15/30F-Cu_*x*_O and 15F both under 1 sun illumination and dark; (d) UV-vis absorption spectrum of 15/30F-Cu_*x*_O and 15F.

A two-electrode SRFB was used to perform photocurrent density–time (*j*–*t*) measurements and investigate the PEC performance of the photoanodes under 1 sun illumination. The NaOH treatment time and the thickness of sputtered Cu have been optimized, identifying 15/30F-Cu_*x*_O as the best p–n junction film (Fig. S5, ESI†). XRD measurements (Fig. 2a, pink curve) confirm that the 15/30F-Cu_*x*_O sample present the hematite phase of α-Fe_2_O_3_ and a mixture of Cu_2_O–CuO (Cu_*x*_O). The sharp diffraction peaks at 2*θ* of 33.1° indicate the (104) plane of the rhombohedral structure of hematite.^48^ The peaks observed at 35.4° and 48.8° correspond to the (002) and (202) planes of CuO, while the peaks at 36.6° (111) and 42.2° (200) are characteristic peaks of Cu_2_O.^49^ The rest of the peaks can be attributed to ITO.^50^ Additionally, we observed that excluding the NaOH treatment of the Cu film (sample 15/30F-CuO, see Experimental section) results only in CuO diffraction peaks without any trace of Cu_2_O (Fig. 2a, green curve). From a morphological point of view, scanning electron micrographs (Fig. S6, ESI†) interestingly show that the 15/30F-Cu_*x*_O heterojunction film exhibits a smoother surface and more uniform grains than the control hematite sample 15F. This can be attributed to the formation of Cu_*x*_O during the annealing process of Cu_2_O on top of α-Fe_2_O_3_.

As shown in Fig. 2b, the unbiased photocurrent density for 15/30F-Cu_*x*_O is 0.24 mA cm^−2^, two times higher than the control hematite sample (0.12 mA cm^−2^). Both 15/30F-Cu_*x*_O and 15F show a stable pulse signal with instantaneous photoresponse, indicating their excellent photoactivity. The long-term operation (1 h) of 15/30F-Cu_*x*_O also exhibits remarkable photocurrent stability, with a current retention of approximately 98% (Fig. S7, ESI†), indicating its robust structure as well as enduring photocatalytic stability. Additionally, linear sweep voltammetry (LSV) curves of these two photoanodes were measured in the same set up both under illumination and dark conditions with a sweeping rate of 10 mV s^−1^ (Fig. 2c). Indeed, the photocurrent onset of 15/30F-Cu_*x*_O is observed around −0.42 V *vs.* carbon felt, slightly higher than that of 15F (−0.52 V), owing to the partial sacrifice of oxidation and reduction potentials by the p–n junction as discussed above. Given their consistent dark current onset (0.3 V), the photovoltage (defined here as the potential difference between dark and light current onset) of 15/30F-Cu_*x*_O is smaller than that of 15F as expected. The unbiased photocurrent at 0 V in linear sweep voltammetry of these two photoanodes are in line with *j*–*t* tests, further demonstrating the significantly increased photocatalytic activity of the heterojunction.

When considering solely the photoelectrode components (*i.e.* excluding considerations of battery resistance losses and redox couple reaction activities), the theoretically achievable photocurrent is decided by the light absorption and the charge carriers transfer from the bulk to the electrode/electrolyte interface of semiconductors.^41^ The optical response of the bare α-Fe_2_O_3_ film was measured and compared with that of the 15/30F-Cu_*x*_O in Fig. 2d. The UV absorption (340–400 nm) increases from 15F to 15/30F-Cu_*x*_O, while the visible light absorption (400–600 nm) of 15F is slightly higher than 15/30F-Cu_*x*_O. Overall, the total light absorption of the 15F and 15/30F-Cu_*x*_O samples in the range 340–700 nm is 0.27 and 0.25 respectively. As these values are comparable, the primary factor influencing photocurrent becomes the photogenerated electron–hole separation process, indicating that the 15/30F-Cu_*x*_O p–n junction exhibits better charge transfer than 15F. As a comparison, we tested thicker hematite films (30F and 50F) with higher absorption as well as their p–n junction counterparts (Fig. S8–S10, ESI†). Yet, in all cases due to the limited charge mobility within hematite,^21^ the measured photocurrent was lower than for the 15/30F-Cu_*x*_O film. Electrochemical Impedance Spectroscopy (EIS) analysis (Fig. S11, ESI†) further confirmed the photocurrent measurements. The internal charge transfer resistance (*R*_sc_) of 15/30F-Cu_*x*_O is 1530 Ω, significantly lower than that of 15F (5430 Ω), suggesting efficient charge carrier transport within the bulk facilitated by the p–n junction. Additionally, the charge transfer resistance at the interface (*R*_ct_) decreases from 15F (4060 Ω) to 15/30F-Cu_*x*_O (1050 Ω), indicating faster photooxidation reaction dynamics at the 15/30F-Cu_*x*_O/ferrocyanide interface compared to the 15F/ferrocyanide interface. Hence, these two factors contribute to the higher photocurrent density of the planar 15/30F-Cu_*x*_O p–n junction.

**Fig. 3:**
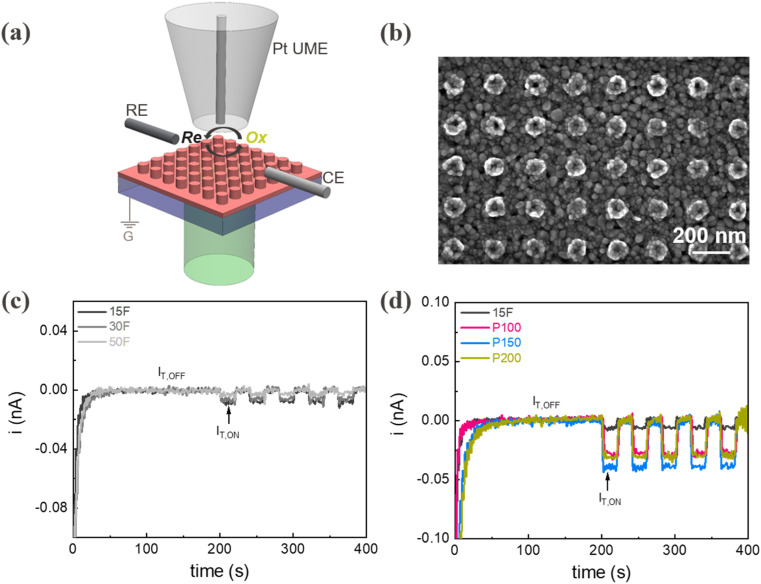
(a) Schematic of light-assisted scanning electrochemical microscopy (photo-SECM) set-up, with reference electrode (RE), counter electrode (CE), reductant (Re) and oxidant (Ox); (b) SEM of P150; SECM to probe α-Fe_2_O_3_ (c) films (F) and (d) nanopillar arrays (P) under white light illumination (80 mW cm^−2^); *I*_T,ON_ is the current under light and *I*_T,OFF_ is the dark current.

To improve charge transfer in thicker hematite films that exhibit higher light absorption (Fig. S8, ESI†), we explored the impact of nanoengineering strategies, which increase the available surface area and reduce the charge transport distances. Indeed, nanoscale patterning of a high-index semiconducting catalyst can create resonant interactions between the nanometer-scale semiconductor and light (Mie modes), which are strongly dependent on the dimensions and periodicity of the structure.^51–53^ Specifically, we realized arrays of Fe nanopillars with well-defined diameter, *D*, and periodicity, *P*, which were subsequently oxidized to obtain α-Fe_2_O_3_ nanopillars. A thin (∼10 nm) α-Fe_2_O_3_ film was left to cover the ITO substrate (see Fig. S1b† and Experimental section). Electromagnetic simulations (COMSOL Multiphysics®) were used to quantify the impact of *D* on the absorption spectrum of α-Fe_2_O_3_ nanopillar arrays based on a 30 nm thick iron (*P* = 300 nm). We observe that, despite the reduction in overall absorbing material, nanostructuring allows the excitation of optical resonance modes that entail a high absorption level (Fig. S9b, ESI†). In order to explore the optimal balance between optical performance and electron transfer, α-Fe_2_O_3_ nanopillar arrays with diameters of 100, 150, and 200 nm (named as P100, P150, and P200 respectively) were patterned *via* e-beam lithography over an area of approximately 100 × 100 μm^2^. The SEM top-view images of nanopillar arrays with different diameters are reported in Fig. 3b and S12a–f (ESI),† showing the increase in the pillar diameter upon annealing, due to oxygen incorporation. Microscale absorption measurements^43^ of the different arrays (Fig. S13, ESI†) were consistent with simulations and showed a minimal decrease in absorption compared to the unpatterned film.

To investigate the effect of nanopatterning on the photocatalytic performance of the α-Fe_2_O_3_ photoanodes, we performed photo-SECM on both films and nanopillar array structures in a 4 mM Fe(CN)_6_^4−^ and 0.4 M NaOH electrolyte solution under white light illumination. The reductively biased Pt UME tip in our experiments locally detects the photo-generated oxidant species, *i.e.* Fe(CN)_6_^3−^ (Fig. 3a). For all the measurements, we controlled the tip to substrate distance by monitoring tip current *versus* distance and positioning the tip at a distance 3 μm higher than the 30% offset value^54^ (Fig. S14, ESI†). Fig. 3c shows the time trace of the tip current for the α-Fe_2_O_3_ films having different thicknesses when the light is modulated on and off at the same power density. Analyzing the *I*_T,ON_/*I*_T,OFF_ values shows that the photocatalytic activity of the film structures decreases by increasing the film thickness from 15F to 30F, and then to 50F. This is in agreement with the lower charge transfer rates observed in the SRFB photoresponse as film thickness increases (Fig. S10a, ESI†). Comparing the *I*_T,ON_/*I*_T,OFF_ values for the α-Fe_2_O_3_ nanopillar arrays (Fig. 3d) with the best performing film structure (15F) shows a significant enhancement (more than 5 folds) in photocatalytic activity, not achievable by only decreasing the film thickness. Most importantly, the results show that the P150 α-Fe_2_O_3_ nanopillar array has the highest photocatalytic activity among all the samples, realizing an optimum combination of surface area, light absorption, and charge transport dynamics.^31^ Based on these local PEC results, we chose 150 nm as the optimum Fe nanopillar diameter for realizing centimeter-scale, nanostructured α-Fe_2_O_3_/Cu_*x*_O photoanodes for SRFBs.

**Fig. 4:**
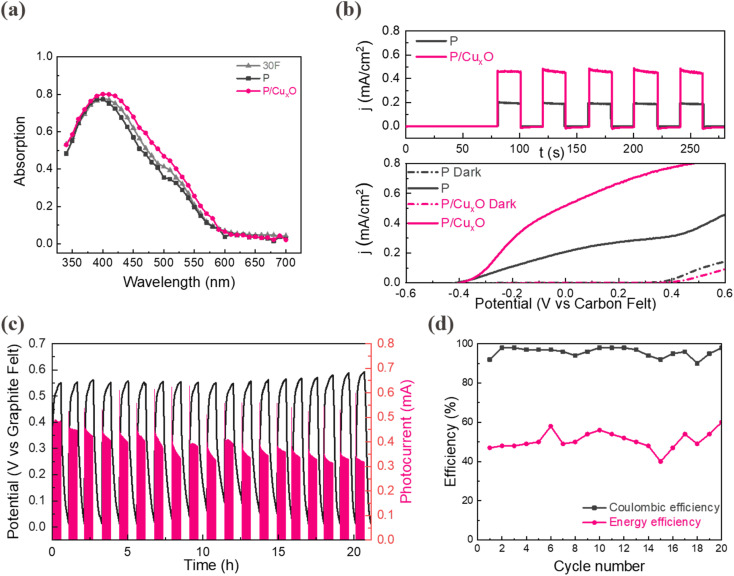
(a) UV-vis absorption spectrum of nanostructures and 30F; (b) photoresponse behavior for P/Cu_*x*_O and P with 20 s light on–off; linear sweep voltammetry curve for P/Cu_*x*_O and P both under 1 sun illumination and dark; (c) representative photocharge–discharge cycling behavior with a cut-off potential set to 0–0.58 V and unbiased photocurrent using P/Cu_*x*_O photoanode; (d) coulombic and energy efficiency of photocharge/discharge curve.

The nanostructured α-Fe_2_O_3_/Cu_*x*_O (P/Cu_*x*_O) photoanode was fabricated employing a combination of nanoengineering and heterojunction design, as illustrated in Fig. S1c (ESI).† A Langmuir–Blodgett (LB) technique, rather than e-beam lithography, was exploited to fabricate nanopatterns over cm-scale samples.^39^ A nanostructured α-Fe_2_O_3_ (P) without copper coating nor NaOH treatment was also fabricated as a control sample. The addition of the Cu_*x*_O layer results in distinct morphological changes. Specifically, the P/Cu_*x*_O exhibits nanorod bundles-like structures on the surface which are absent on the P sample (Fig. S15, ESI†). Optically, the P sample exhibits a total light absorption (340–700 nm range) of 0.34, similar to the unpatterned 30F film (0.36) while the P/Cu_*x*_O sample exhibits a small but broadband absorption increase that results in a larger total absorption of 0.39 (Fig. 4a).

Photoelectrochemically, the nanostructured α-Fe_2_O_3_ (P) shows a photocurrent density of 0.2 mA cm^−2^ (Fig. 4b), nearly two folds higher than the best thin film sample, 15F, consistent with the SECM analysis. With the addition of the p–n junction, which improves both light harnessing and carrier transport, the P/Cu_*x*_O sample exhibits an outstanding photocurrent density of 0.46 mA cm^−2^ as well as a good stability (Fig. S16, ESI†). Fig. 4b illustrates the variation of photocurrent density with applied voltage. At 0 V, the photocurrent density for P/Cu_*x*_O and P are 0.49 mA cm^−2^ and 0.21 mA cm^−2^, respectively. The higher photocurrents compared to the photoresponse tests may arise from the transient current generated by the double-layer capacitance under voltage changes as well as the trapped photogenerated holes due to the existence of detrimental surface states at the electrode–electrolyte interface.^55^ Interestingly, the similar photovoltage (0.75 V) of both photoanodes suggest that the nanostructured p–n junction does not compromise its capability to drive these redox couples. EIS studies were also carried out under illumination to gain insight into the effect of the p–n junction on PEC redox oxidation reaction. The same electrical circuit was used as a model to fit the photooxidation process in the photoanodes (Fig. S17, ESI†). In contrast to P, the P/Cu_*x*_O exhibits significantly lower values of *R*_sc_ (500 Ω) and *R*_ct_ (757 Ω), indicating that the surface coverage of an additional Cu_*x*_O layer can improve the charge separation inside bulk α-Fe_2_O_3_ as well as reduce the charge extraction barrier to create a facile carrier pathway at the electrode/electrolyte interface. Additionally, the space-charge capacitance (*C*_sc_) decreases from P/Cu_*x*_O (1.4 × 10^−3^ F) to P (0.03 × 10^−3^ F), implying the broadening of the depletion layer of the P photoanode and thus much inferior carrier mobility.^56–58^

After demonstrating the photooxidation reaction activity and stability of the P/Cu_*x*_O photoanodes, the photocharge/discharge test was finally performed using a fully integrated SRFB. The photocharge/discharge curves of the SRFB for the initial 20 cycles (approximately 22 h) are shown in Fig. 4c. The average photocurrent density is equal to 0.42 mA cm^−2^ (0.33 mA when considering the active area) under 1 sun illumination, with a discharging current applied of −0.4 mA. This integrated system exhibits a stable and high coulombic efficiency of around 90–98% and a stable average energy efficiency around 50% as shown in Fig. 4d. Overall, the P/Cu_*x*_O-based SRFB achieves a stable solar-to-chemical efficiency of 0.35% and an average solar-to-output energy efficiency of 0.18% over 20 cycles, which is a significant progress for unassisted α-Fe_2_O_3_-based SRFB.

## Conclusion

In summary, the synergistic design of nanostructuring and engineered heterojunction for photoelectrodes was realized to improve the SRFB performance. Firstly, we conducted a comprehensive investigation through band alignment engineering and various electrochemical techniques, elucidating the enhancement of carrier transport both within the bulk photoelectrode and at the photoelectrode/electrolyte interface facilitated by the Cu_*x*_O–Fe_2_O_3_ p–n junction rather than CuO–Fe_2_O_3_. Secondly, leveraging the localized measurements of SECM, we are able to utilize microscale samples to seamlessly explore the nanostructure sizes effect on the photoelectrodes performance. Nanoengineering not only enables the manipulation of the optical properties of the photoelectrodes (including light trapping and Mie resonance), but also provides additional reactive sites, effectively mitigating losses attributable to charge recombination. Indeed, by combining these two strategies, the nanorod bundles-like P/Cu_*x*_O exhibits the highest unbiased photocurrent density (0.46 mA cm^−2^) as well as good stability for unassisted α-Fe_2_O_3_-based SRFB. The average STC efficiency during photocharge process reaches 0.35% and the solar-to-output energy efficiency of the as-designed photoanode is 0.18%, a performance level previously achieved in hematite systems only with the assistance of external solar cells. Overall, the straightforward photoanode preparation process, the earth-abundant material choice along with the remarkable performance should be promising for the practical application of the solar rechargeable batteries. In addition, further advancements in the SRFB system can be anticipated, particularly in terms of microfluidic design to enhance mass transport and nanophotonic engineering to improve charge transfer and optical performance. These SRFB design concepts may open new avenues for reaching highly efficient solar redox flow batteries.

## Conflicts of interest

The authors declare no conflict of interest.

## Supplementary Material

TA-013-D4TA06302C-s001

## Data Availability

The data supporting this article have been included as part of the ESI.†
