# Supplementary material for: Nanostructured Fe2O3/CuxO heterojunction for enhanced solar redox flow battery performance
Source: J Mater Chem A Mater. 2024 Nov 27;13(2):1320–9. doi: 10.1039/d4ta06302c (PMC11622009; doi:10.1039/d4ta06302c)
Supplement: TA-013-D4TA06302C-s001 [file TA-013-D4TA06302C-s001.pdf]

Supplementary Information (SI) for Journal of Materials Chemistry A.
This journal is © The Royal Society of Chemistry 2024

Nanostructured Fe_2O_3/Cu_xO Heterojunction for Enhanced Solar Redox Flow Battery Performance

*Jiaming Ma[1], Milad Sabzehparvar[1], Ziyan Pan[1], Giulia Tagliabue[1]**

[1] Laboratory of Nanoscience for Energy Technologies (LNET), STI, École Polytechnique Fédérale de Lausanne, 1015 Lausanne, Switzerland

**Corresponding Author. Email: giulia.tagliabue@epfl.ch*

Table of Contents

Supporting Information 1 – Comparison of hematite SRFBs performance 2
Supporting Information 2 – Schematic of samples preparation 3
Supporting Information 3 – Schematic of SRFB structure 4
Supporting Information 4 – Cross section TEM-EDX measurements 5
Supporting Information 5 – Band alignment measurements 6
Supporting Information 6 – Investigation of NaOH treatment time and Cu film thickness 7
Supporting Information 7 – Scanning electron microscope of planar sample 8
Supporting Information 8 – Stability of planar sample 9
Supporting Information 9 – Optical performance of hematite films with different thicknesses 10
Supporting Information 10 – Optical simulation 11
Supporting Information 11 – Photoresponse testing of various planar p-n junctions 12
Supporting Information 12 – Electrochemical impedance spectroscopy analysis 13
Supporting Information 13 – SEM and optical microscopy image of different patterns 14
Supporting Information 14 – Microscale absorption measurements 15
Supporting Information 15 – Tip approaching curve 16
Supporting Information 16 – SEM for nanostructures 17
Supporting Information 17 – Photoresponse and stability for nanostructures 18
Supporting Information 18 – EIS analysis for nanostructures 19
Reference 20

Supporting Information 1 – Comparison of hematite SRFBs performance

Photoanode	Redox couples	j (mA/cm^2)	STC	SOEE
P/Cu_xO (This work)	$Na_4Fe(CN)_6$ in NaOH/ AQDS in NaOH	0.46	0.35%	0.18%
α-Fe_2O_3 porous NPs[1]	$Na_4Fe(CN)_6$ in NaOH/ AQDS in NaOH	0.22	0.17%-0.23%	0.11%
Polyaniline coated α-Fe_2O_3 thin-film[2]	$Na_4Fe(CN)_6$ in NaOH/ AQDS in NaOH	0.04	0.03%	---
α-Fe_2O_3 thin-film/ DSSC[3]	I_2 in NH_4I/ AQDS in NH_4I	0.03	0.03%	---
		0.18	0.15%	
α-Fe_2O_3 thin-film/ DSSC[4]	$Na_4Fe(CN)_6$ in NaOH/ AQDS in NaOH	0.04	0.03%	---
		0.39	0.29%	0.15%
α-Fe_2O_3 nanocubes/ DSSC[4]	$Na_4Fe(CN)_6$ in NaOH/ AQDS in NaOH	0.04	0.03%	---
		1.36	1%	0.44%

Table S1. Solar Redox Flow Cell based on α-Fe_2O_3 photoanode reported in literature and their performance, including photocurrent density, solar-to-chemical efficiency (STC) and solar-to-output energy efficiency (SOEE). Cells filled in grey refer to performance of dye-sensitized-solar-cell (DSSC) assisted devices while white cells refer to unassisted devices.

Supporting Information 2 – Schematic of samples preparation

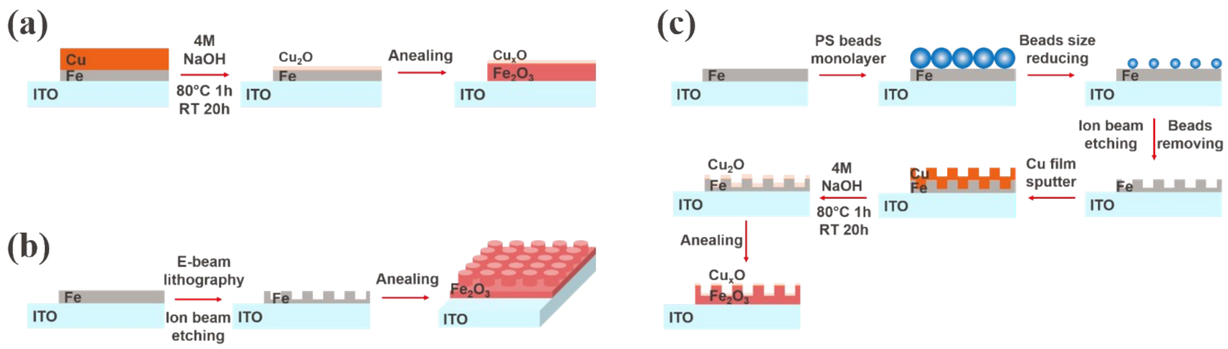

Figure S1. (a) Schematic of the synthesis of α-Fe_2O_3/Cu_xO film photoanodes; (b) Schematic of the synthesis process of α-Fe_2O_3 nanopillar array; (c) Schematic of the synthesis of nanostructured α-Fe_2O_3/Cu_xO photoanode. All the materials were sputtered on the ITO substrate.

Table S2. Summary of sample preparation process.

Sample type	Sputtered Fe (nm)	Sputtered Cu (nm)	NaOH	Annealing	Sample
Planar Fe_2O_3	15	0	NO	YES	15F
	30	0	NO	YES	30F
	50	0	NO	YES	50F
Planar Fe_2O_3-CuO	15	30	NO	YES	15/30 F-CuO
Planar Fe_2O_3-Cu_xO	15	30	YES	YES	15/30 F-Cu_xO
	30	30	YES	YES	30/30 F-Cu_xO
	50	30	YES	YES	50/30 F-Cu_xO
Nanopatterned Fe_2O_3	30	0	NO	YES	P100<br>P150<br>P200
Nanostructured Fe_2O_3	30	0	NO	YES	P
Nanostructured Fe_2O_3-Cu_xO	30	30	YES	YES	P/Cu_xO

Supporting Information 3 – Schematic of SRFB structure

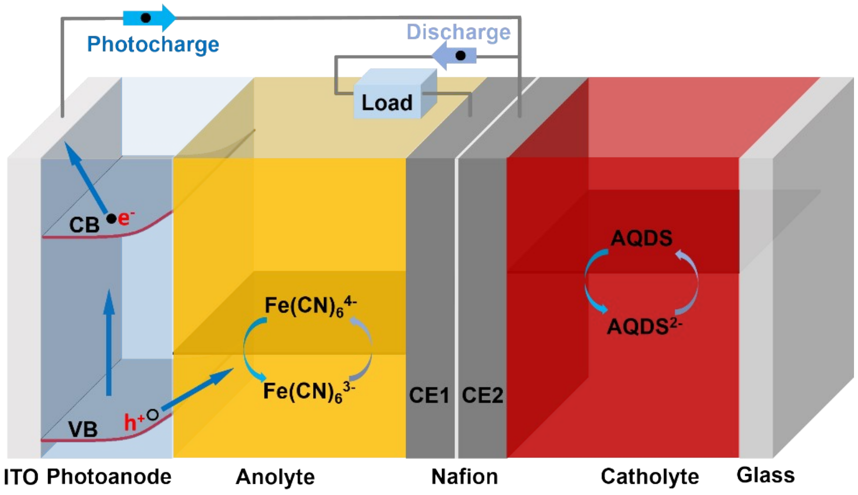

Figure S2. Schematic of the SRFB used in this work, CE1 and CE2 are carbon felt 1 and carbon felt 2.

This integrated SRFB system consists of one photoelectrode and two carbon felt electrodes. For the two-electrode test, the photoanode in the anolyte was used as the working electrode, with a 39 AA carbon felt in the catholyte serving as the counter electrode (CE2). In the photocharge-discharge tests, the photocharging process involved the photoanode as the working electrode and CE2 as the counter electrode. During discharge, the CE2 in the catholyte acted as the working electrode, while another 39 AA carbon felt in the anolyte was used as the counter electrode (CE1). Thus, the complete photocharge-discharge test is effectively a three-electrode system.

Supporting Information 4 – Cross section TEM-EDX measurements

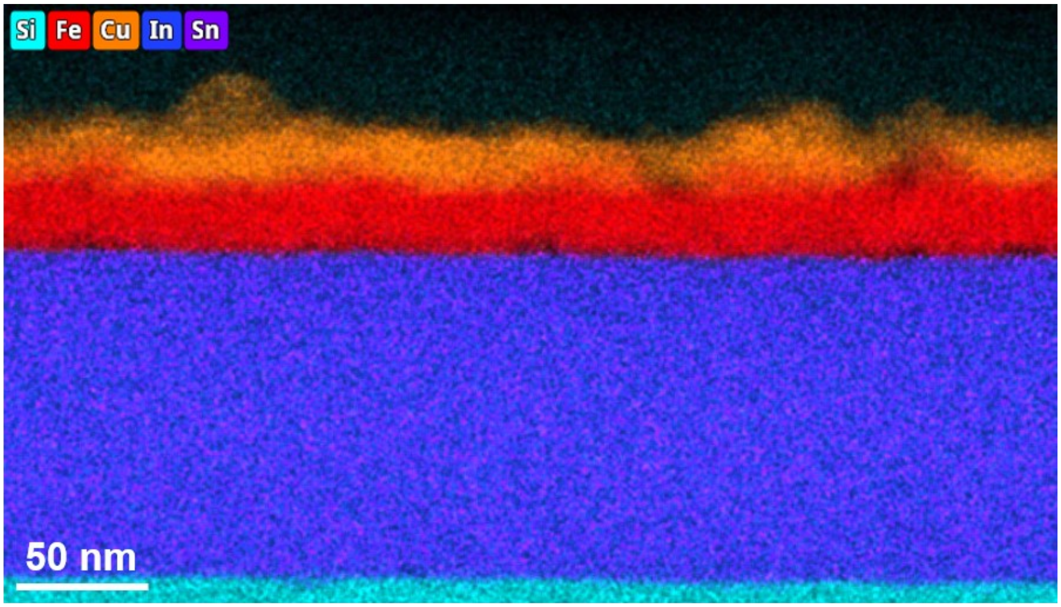

Figure S3. Cross section TEM-EDX of 15/30 F-Cu_xO, Tin in purple, iron in red and Cu in orange.

The cross section comes from a focused ion beam (FIB) lamellar sample with carbon protected on top. It can be seen that the 15 nm film Fe results in 30 nm α-Fe_2O_3 after annealing, while the average thickness of the Cu_xO is ~15 nm.

Supporting Information 5 – Band alignment measurements

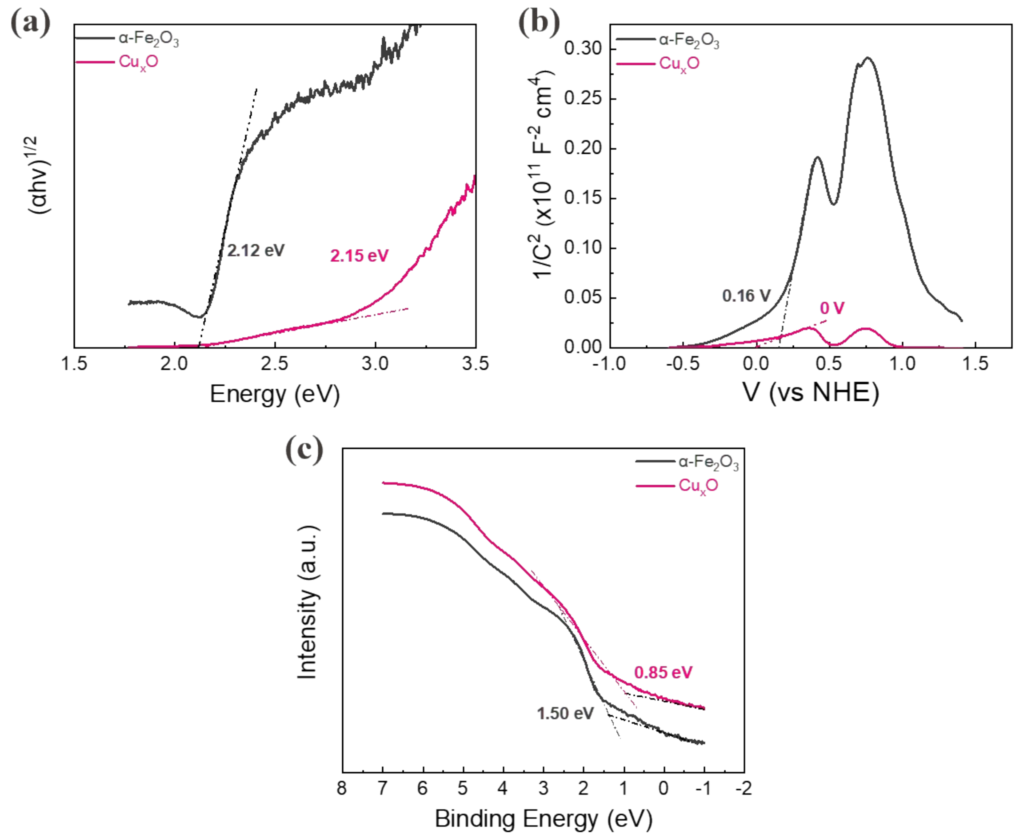

Figure S4. (a) Tauc plots, (b) Mott-schottky test and (c) UPS test of α-Fe_2O_3 and Cu_xO to get bandgap, work function and valence band maxima (VBM); respectively.

The band gap value is estimated by identifying the intersection point between the linear portion of Tauc's plots and the X-axis. These plots of the absorption coefficient (α) were obtained using a UV-visible spectrophotometer (Shimadzu, UV-2600i) within the wavelength range of 300−800 nm. Mott−Schottky (MS) measurements were conducted under dark conditions using a three-electrode homemade cell via CHI 760E potentiostat. The photoanode served as the working electrode, while a Pt foil (99.99% purity, Alfa Aesar) acted as the counter electrode. Additionally, a leakage-free Ag/AgCl electrode (Innovative Instruments, Inc) was utilized as the reference electrode. The experiment was performed within a potential range of −0.8 to 1.2 V vs Ag/AgCl under anolyte, employing an applied frequency of 1 kHz to investigate the intrinsic electrical properties. The flat band potential (F_{fb}) value can be determined using the following equation[5]:

$$C^{-2} = \frac{2}{q\epsilon_0\epsilon_s N_D}\left[V - F_{fb} - \frac{KT}{q}\right]$$

Supporting Information 6 – Investigation of NaOH treatment time and Cu film thickness

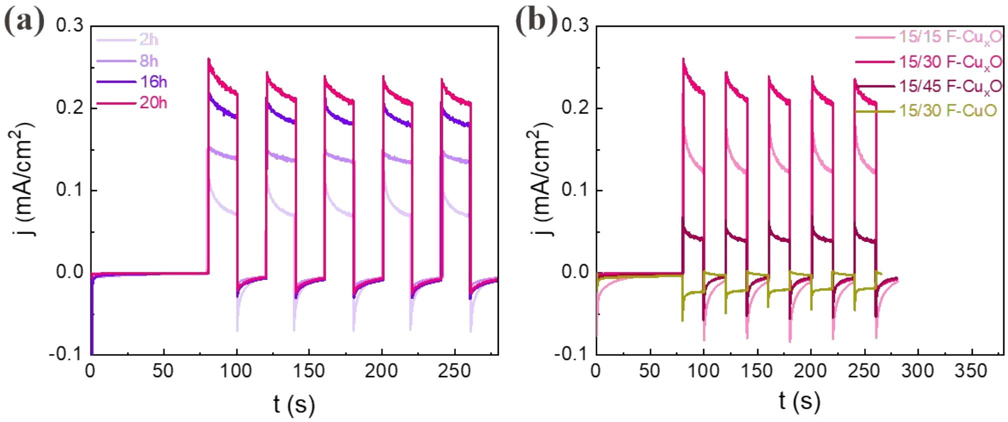

Figure S5. (a) Photoresponse behavior of 15/30 F-Cu_xO with different NaOH treatment time; (b) Photocurrent performance of 15F-based heterojunctions with varying thicknesses of deposited Cu films subsequently treated for 20 h in NaOH.

Supporting Information 7 – Scanning electron microscope of planar sample

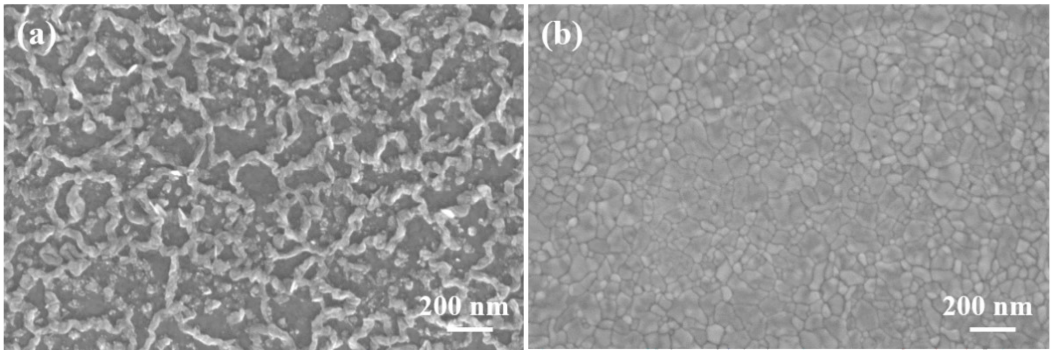

Figure S6. SEM of (a) 15F and (b) 15/30 F-Cu_xO.

Supporting Information 8 – Stability of planar sample

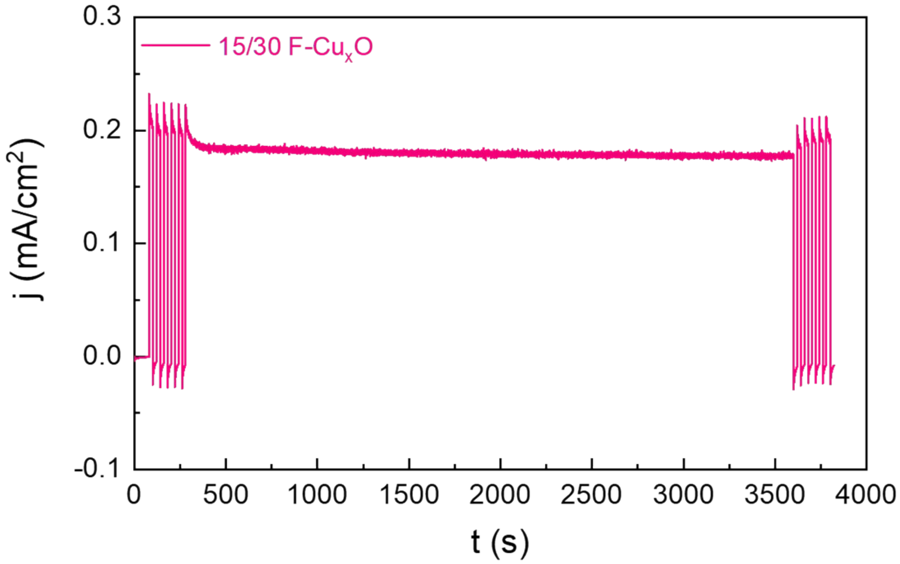

Figure S7. Stability test (1h) of 15/30 F-Cu_xO with photoresponse before and after.

Supporting Information 9 – Optical performance of hematite films with different thicknesses

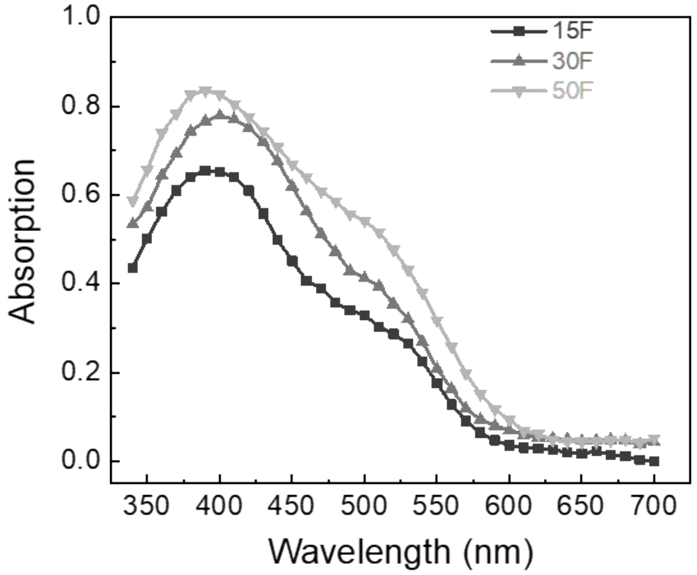

Figure S8. UV-Vis absorption spectrum of 15F, 30F and 50F.

Supporting Information 10 – Optical simulation

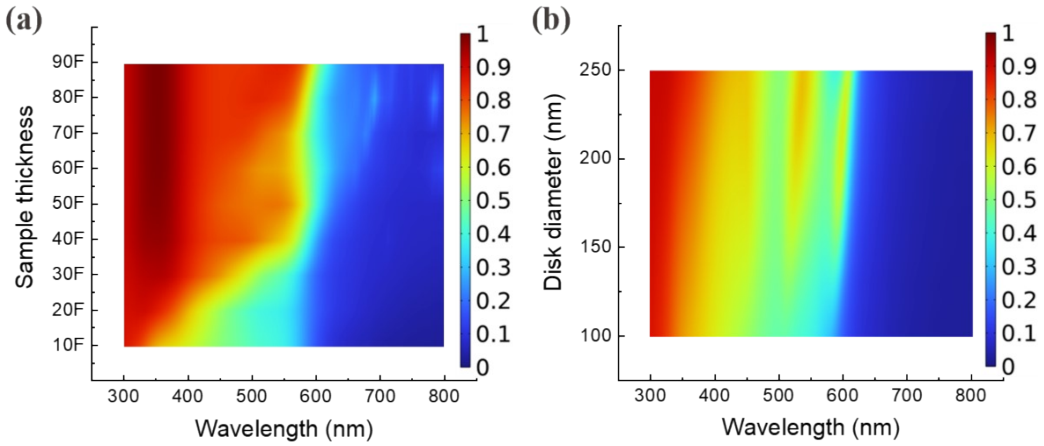

Figure S9. Simulated 2D absorption spectra of the (a) α-Fe_2O_3 film with different thickness and (b) 30F-based α-Fe_2O_3 nanopillar arrays with varying diameters under a periodicity of 300 nm.

Supporting Information 11 – Photoresponse testing of various planar p-n junctions

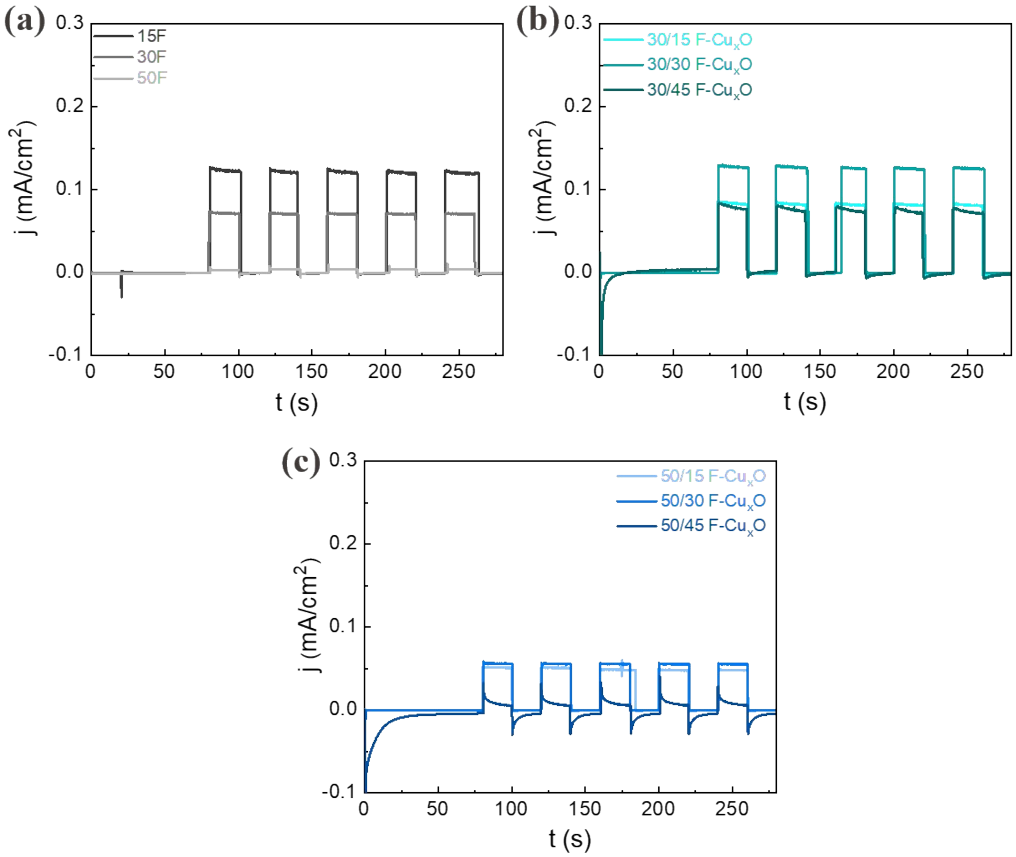

Figure S10. (a) The influence of α-Fe_2O_3 thickness on the photoelectrochemical performance; Photoresponse of (b) 30F-based and (c) 50F-based heterojunctions with varying thicknesses of deposited Cu films treated for 20 h in NaOH.

As a comparison, we measured the absorption spectra and photochemical activity of pure hematite films with different thicknesses (Figure S8 and Figure S10) under back illumination in ambient air conditions as described in Experimental Section. The measured absorption curves align well with numerical calculations (Figure S9a). The light absorption demonstrates a notable increase from 15F to 30F, followed by saturation upon reaching 50F. Yet, due to the limited charge mobility within hematite[6], electron-hole recombination significantly constrains the performance of the photoanodes. The photocurrent density decreases from 15F (0.12 mA/cm^2) to 30F (0.07 mA/cm^2) and experiences a significant decrease at 50F (0.01 mA/cm^2) thereafter (Figure S10a). Despite efforts to construct p-n junctions to improve charge transfer, the best samples obtained with thicker hematite films only exhibit a photocurrent density of 0.14 mA/cm^2 and 0.06 mA/cm^2 for 30/30 F-Cu_xO and 50/30 F-Cu_xO, respectively (Figure S10b, c)

Supporting Information 12 – Electrochemical impedance spectroscopy analysis

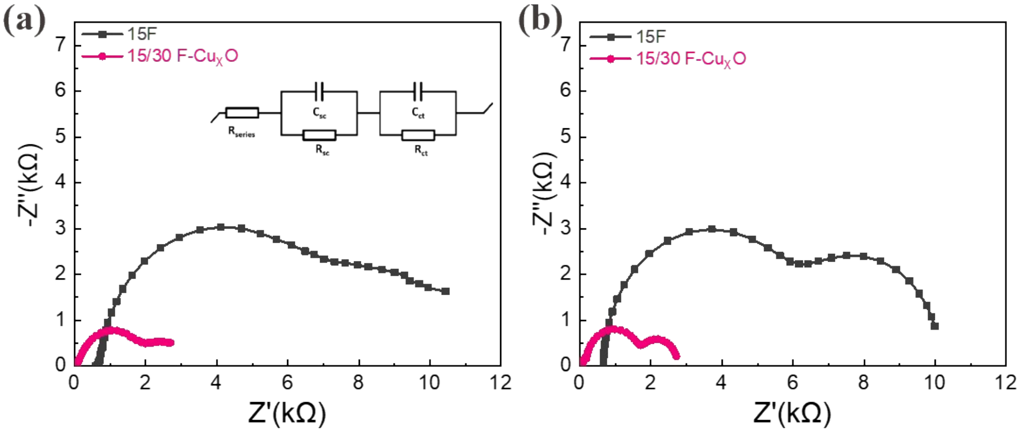

Figure S11. The (a) measured and (b) fitting EIS results of 15F and 15/30 F-Cu_xO. The inset is the equivalent electrical circuit.

Electrochemical Impedance Spectroscopy (EIS) analysis was performed with frequency ranging from 10 kHz to 10 mHz under 1 sun illumination to further investigate the mechanisms that occur at the photoanodes. The electrical circuit (the inset of Figure S11a) was used as a model to fit the PEC redox couple oxidation reaction process in the photoanodes. Where R_{series} represents the series resistance, which includes the resistance at the ITO/photoanode interface, the ionic conductivity in the electrolyte and the external contact resistance; R_{sc} and C_{sc} are models of charge transfer behavior in the internal semiconductor, while R_{ct} and C_{ct} are the analogous reactions that take place at the semiconductor/electrolyte interface. The fitting Nyquist plots are shown in Figure S11b, which is similar to the measured EIS results.

Supporting Information 13 – SEM and optical microscopy image of different patterns

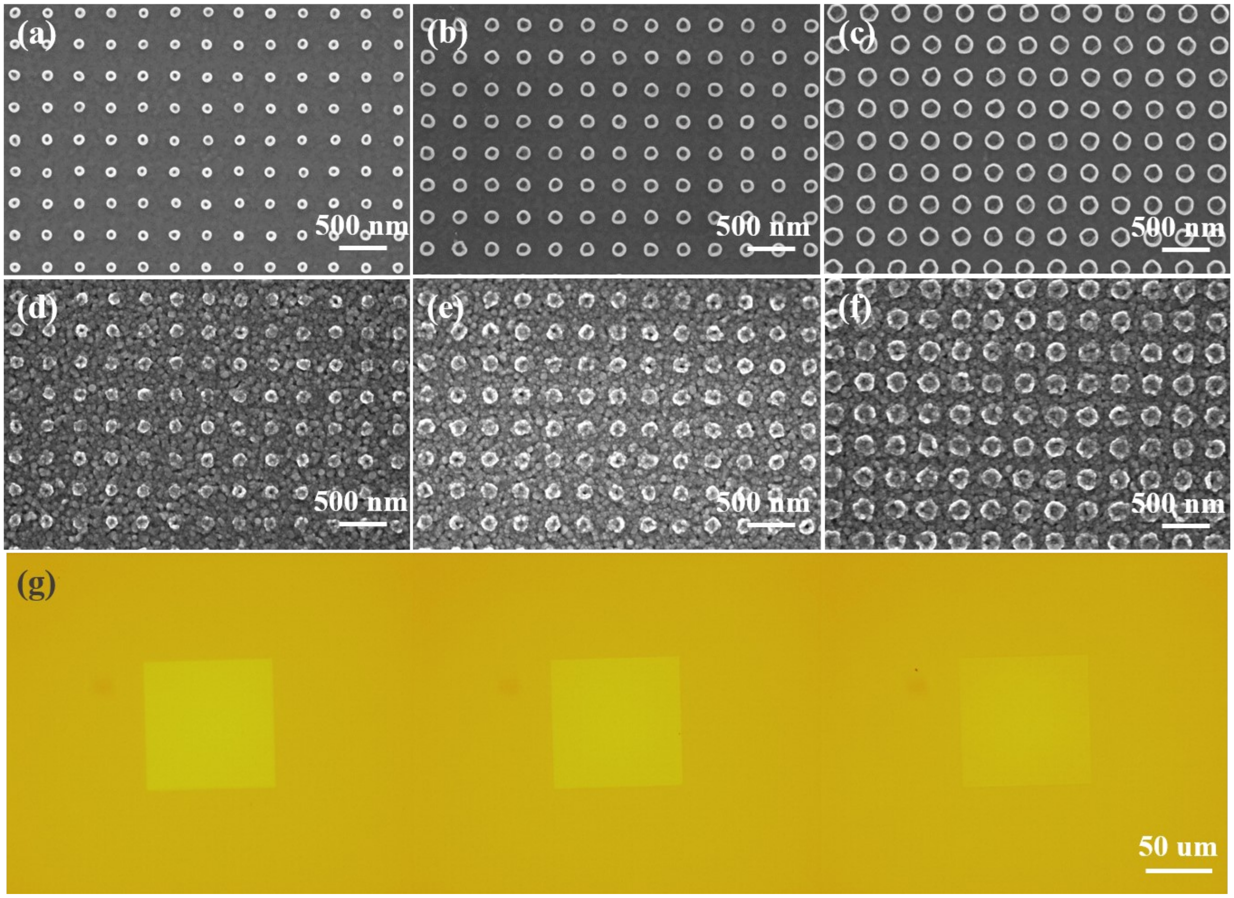

Figure S12. SEM of (a, d) P100, (b, e) P150 and (c, f) P200 before and after annealing; (g) Optical microscopy image of the α-Fe_2O_3 nanopillar arrays.

The corresponding optical, white-light transmission images (Figure S12g) exhibit the different optical appearances of the three patterns, providing a direct way to observe the tunability of the optical properties with varying diameters.

Supporting Information 14 – Microscale absorption measurements

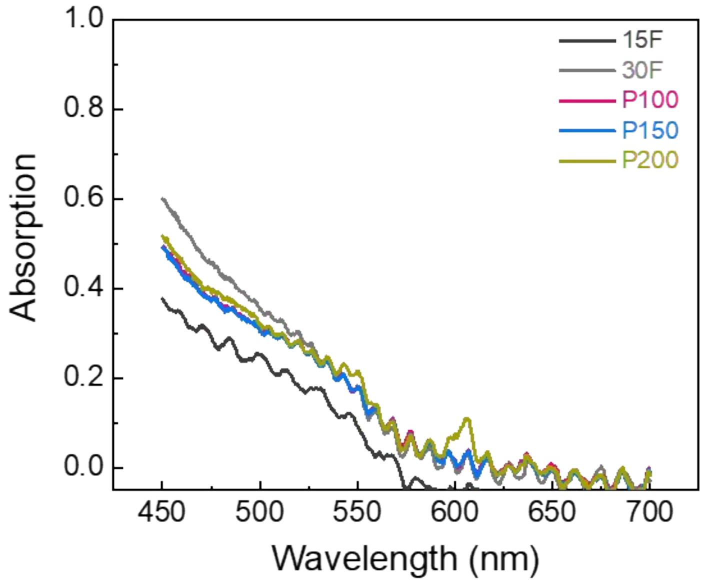

Figure S13. Microscale absorption measurements of the fabricated α-Fe_2O_3 nanopillar arrays as well as α-Fe_2O_3 films.

The absorption depth for incident photons of hematite[7] is 40-100 nm for wavelengths ranging from 450 to 550 nm. A significant enhancement in broadband light absorption can be observed from Figure S13 as the thickness of the thin film sample increases from 15F (~30 nm hematite) to 30F (~60 nm hematite). However, the hole diffusion length limitation for hematite[8] necessitated a sacrifice in the thickness of the photoanode. Nanoengineering could potentially serve as a solution strategy to balance optical performance and charge carrier transport. It is worth noting that all the 30F-based nanopillar arrays (even those with only a 10 nm thickness of continuous α-Fe_2O_3 thin film underneath) exhibit comparable optical absorption to the 30F sample, significantly stronger than the 15F sample. This enhancement could be attributed to the light-trapping effect of the nanopillar arrays[9]. Additionally, P200 enables sunlight to induce optical resonances at 550 nm and 600 nm, consistent with our simulation findings (Figure S9b). This phenomenon can enhance the light intensity within the photoanode[10].

Supporting Information 15 – Tip approaching curve

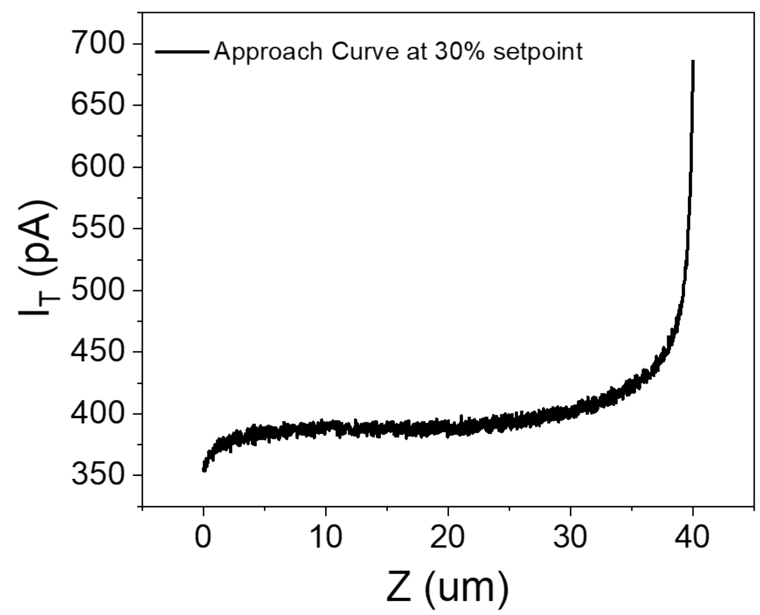

Figure S14. Tip approach curve. Experimental current–distance curves (points) obtained with the tip approaching the hematite film part of the substrate. The solution contained 4 mM $Fe(CN)_6^{4-}$ and 0.4 M NaOH. Tip potential E_T=0.4 V vs. Ag/AgCl and an unbiased grounded substrate in dark.

Supporting Information 16 – SEM for nanostructures

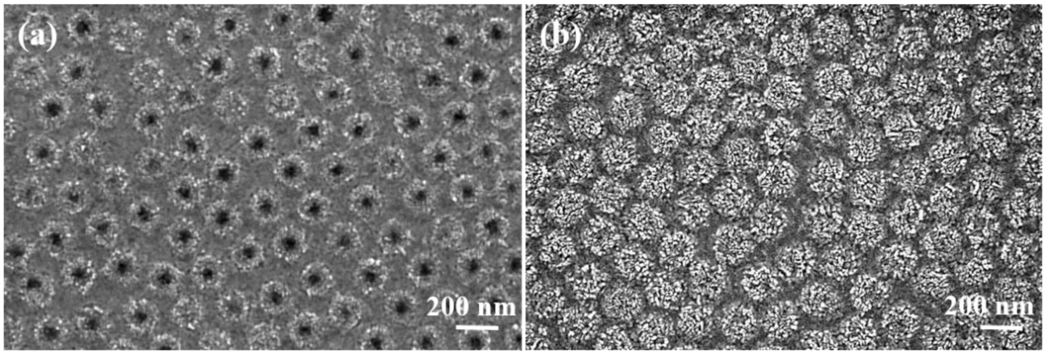

Figure S15. SEM of (a) P and (b) P/Cu_xO.

Supporting Information 17 – Photoresponse and stability for nanostructures

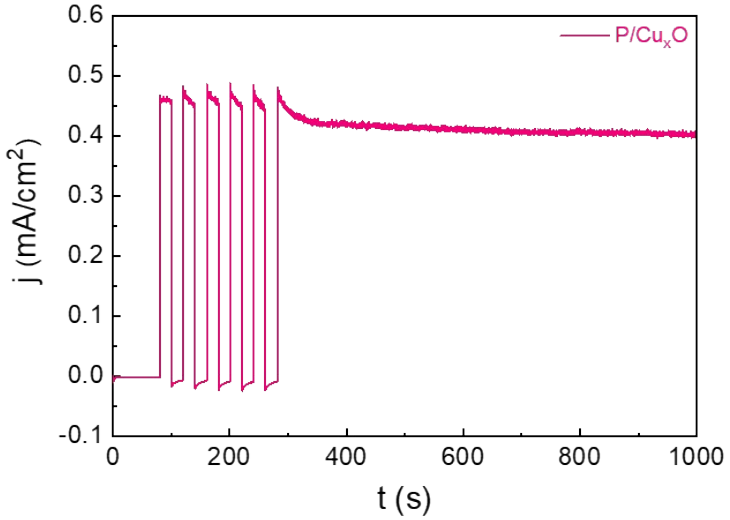

Figure S16. Stability of j-t for P/Cu_xO.

Supporting Information 18 – EIS analysis for nanostructures

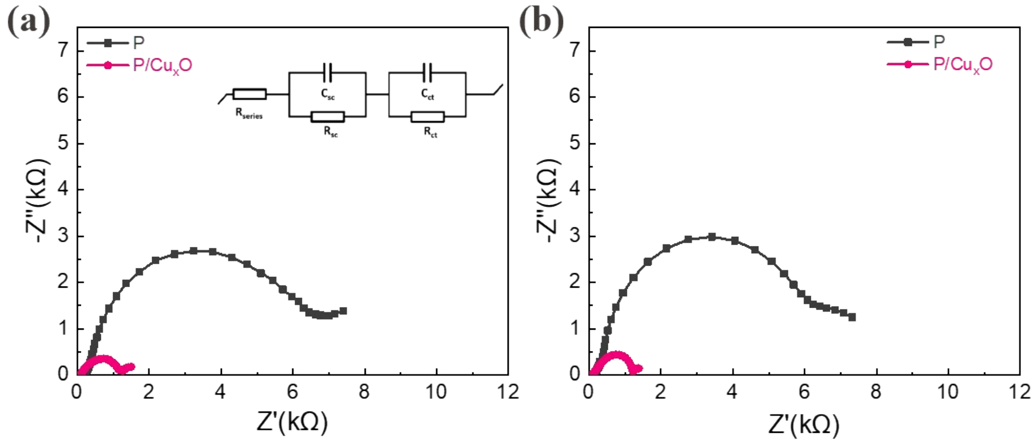

Figure S17. The (a) measured and (b) fitting EIS results of α-Fe_2O_3 P and P/Cu_xO. The inset is the equivalent electrical circuit.

By using the same equivalent electrical circuit as Figure S11, we can get the R_{series}, R_{sc} , R_{ct} and C_{sc} for α-Fe_2O_3 P is 160 Ω, 4075 Ω, 2028 Ω and 0.03×10^{-3} F, respectively. The R_{series}, R_{sc} , R_{ct} and C_{sc} for P/Cu_xO is 90 Ω, 500 Ω, 757 Ω and 1.4×10^{-3} F, respectively.

Reference

(1) Ma, J.; Pan, Z.; Tagliabue, G. High-Performance Hematite Photoanodes for Unassisted Recharging of Solar Redox Flow Battery. *Sol. RRL* **2024**, *8* (17), 2400477. https://doi.org/10.1002/solr.202400477.

(2) Wedege, K.; Azevedo, J.; Khataee, A.; Bentien, A.; Mendes, A. Direct Solar Charging of an Organic–Inorganic, Stable, and Aqueous Alkaline Redox Flow Battery with a Hematite Photoanode. *Angew. Chem. Int. Ed.* **2016**, *55* (25), 7142–7147. https://doi.org/10.1002/anie.201602451.

(3) Khataee, A.; Azevedo, J.; Dias, P.; Ivanou, D.; Dražević, E.; Bentien, A.; Mendes, A. Integrated Design of Hematite and Dye-Sensitized Solar Cell for Unbiased Solar Charging of an Organic-Inorganic Redox Flow Battery. *Nano Energy* **2019**, *62*, 832–843. https://doi.org/10.1016/j.nanoen.2019.06.001.

(4) da Silva Lopes, T.; Dias, P.; Monteiro, R.; Vilanova, A.; Ivanou, D.; Mendes, A. A 25 Cm2 Solar Redox Flow Cell: Facing the Engineering Challenges of Upscaling. *Adv. Energy Mater.* **2022**, *12* (5), 2102893. https://doi.org/10.1002/aenm.202102893.

(5) Singh, A. P.; Kodan, N.; Mehta, B. R.; Held, A.; Mayrhofer, L.; Moseler, M. Band Edge Engineering in BiVO4/TiO2 Heterostructure: Enhanced Photoelectrochemical Performance through Improved Charge Transfer. *ACS Catal.* **2016**, *6* (8), 5311–5318. https://doi.org/10.1021/acscatal.6b00956.

(6) Gao, R.-T.; Zhang, J.; Nakajima, T.; He, J.; Liu, X.; Zhang, X.; Wang, L.; Wu, L. Single-Atomic-Site Platinum Steers Photogenerated Charge Carrier Lifetime of Hematite Nanoflakes for Photoelectrochemical Water Splitting. *Nat. Commun.* **2023**, *14* (1), 2640. https://doi.org/10.1038/s41467-023-38343-6.

(7) Gardner, R. F. G.; Sweett, F.; Tanner, D. W. The Electrical Properties of Alpha Ferric Oxide—II.: Ferric Oxide of High Purity. *J. Phys. Chem. Solids* **1963**, *24* (10), 1183–1196. https://doi.org/10.1016/0022-3697(63)90235-X.

(8) Kennedy, J. H.; Frese, K. W. Photooxidation of Water at α - Fe2 O 3 Electrodes. *J. Electrochem. Soc.* **1978**, *125* (5), 709. https://doi.org/10.1149/1.2131532.

(9) Kiani, F.; Sterl, F.; Tsoulos, T. V.; Weber, K.; Giessen, H.; Tagliabue, G. Ultra-Broadband and Omnidirectional Perfect Absorber Based on Copper Nanowire/Carbon Nanotube Hierarchical Structure. *ACS Photonics* **2020**, *7* (2), 366–374. https://doi.org/10.1021/acsphotonics.9b01658.

(10) Kim, S. J.; Thomann, I.; Park, J.; Kang, J.-H.; Vasudev, A. P.; Brongersma, M. L. Light Trapping for Solar Fuel Generation with Mie Resonances. *Nano Lett.* **2014**, *14* (3), 1446–1452. https://doi.org/10.1021/nl404575e.
